# A Novel Multi-Observation System to Study the Effects of Anterior Ocular Inflammation in Zinn’s Zonule Using One Specimen

**DOI:** 10.3390/ijms24076254

**Published:** 2023-03-26

**Authors:** Akira Takahashi, Takeshi Arima, Etsuko Toda, Shinichiro Kobayakawa, Akira Shimizu, Hiroshi Takahashi

**Affiliations:** 1 Department of Ophthalmology, Nippon Medical School, Tokyo 113-8602, Japan; s11-060ta@nms.ac.jp (A.T.);; 2 Department of Analytic Human Pathology, Nippon Medical School, Tokyo 113-8602, Japan; 3 Department of Ophthalmology, Nippon Medical School, Musashikosugi Hospital, Kanagawa 211-8533, Japan

**Keywords:** Zinn’s zonule, matrix metalloproteinase 2, LV-SEM, fibrillar girdle, cornea, alkali burn injury, inflammation

## Abstract

Zinn’s zonule is a fragile and thin tissue, and little is known about its pathogenesis. The aim of this study was to develop an experimental setup for a comprehensive analysis of Zinn’s zonule. Rats were divided into two groups: a control group (*n* = 4) and an alkali injury group (*n* = 4). Seven days after injury, the eyes were enucleated, the anterior eye was dissected and embedded in gelatin, and macroscopic observations were made. The gelatin specimens were then embedded in paraffin and observed in detail by low-vacuum scanning electron microscopy, immunofluorescence, and quantitative reverse transcription polymerase chain reaction (RT-qPCR). The results show qualitative changes in Zinn’s zonules in both macroscopic and microscopic observations. In addition, macrophage infiltration and increased matrix metalloproteinase 2 (MMP2) expression were observed in the injured group, consistent with the RT-qPCR results. The experimental system in this study allowed us to capture the morphological and molecular biological changes of Zinn’s zonule and to gain insight into its pathogenesis. In conclusion, this study presents a new experimental setup for the comprehensive analysis of the rat Zinn’s zonule. The results suggest that this system can be used in the future to study and analyze a variety of paraffin-embedded tissues and specimens.

## 1. Introduction

Zinn’s zonule is the thin tissue that supports the lens and is composed of an extracellular matrix. The fragility of Zinn’s zonule increases the surgical difficulty of cataract surgeries and causes post-operative complications such as lens deviation and drop. Congenital causes of Zinn’s zonule weakness include Marfan syndrome and homocystinuria, while acquired causes include trauma, pseudo-exfoliation syndrome, uveitis, post-vitrectomy, retinitis pigmentosa, and atopy [1,2,3,4,5]. Inflammation is thought to be involved in acquired causes, and inflammatory infiltrating cells such as macrophages have been reported to migrate through the Zinn’s zonule in corneal debris in an injury model [6]. However, there are few reports on the relationship between Zinn’s zonule and inflammation. This may also just be a consequence of the fragility of Zinn’s zonule, which makes it extremely difficult to be observed in an intact manner. In this study, we developed an experimental system to comprehensively observe Zinn’s zonule using a rat cornea alkali burn model for the first time.

## 2. Results

### 2.1. Macroscopic Observation of Inflammatory Changes in Zinn’s Zonule Using Gel-Embedded Specimens

**Figure 1:**
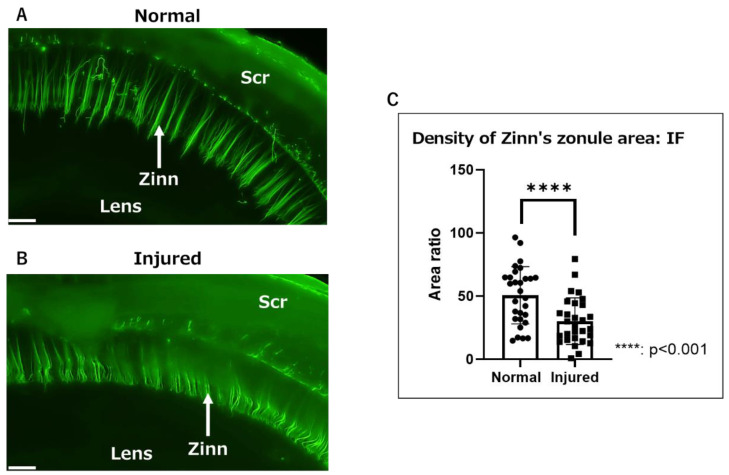
Macroscopic observation of Zinn’s zonule in gel-embedded specimens. (**A**,**B**) Fluorescence images (magnified 40×) of Zinn’s zonule in a rat eye. Zinn’s zonule was stained with anti-MAGP1 (green). (**A**) Zinn’s zonule was clearly visible in the normal group in a three-dimensional view. (**B**) The background staining was stronger and Zinn’s zonule was deflected in the injured group. (**C**) The MAGP1-positive area was measured for 6–8 microscopic fields per specimen. The ratio of Zinn’s zonule area over the area between the lens and scleral wall was calculated to normalize the individual differences among specimens. There was a significant decrease in the fluorescence-positive area in the injured group. Four rat specimens were imaged for the normal and injured groups and the entire Zinn’s zonule was analyzed, for both groups. Zinn: Zinn’s zonule, Scr: sclera, IF: immunofluorescence. Scale bar: 200 μm.

We observed Zinn’s zonule attached to the lens capsule in normal eyes (Figure 1A) and in those with injured cornea on day 7 after alkali burn (Figure 1B). To quantify the inflammatory changes in Zinn’s zonule, microfibril-associated glycoprotein 1 (MAGP1)-positive areas were measured, along with the ratio of the lens scleral area. A significant reduction in the MAGP1-positive area was observed in the injured group compared to the normal group, suggesting a decrease in microfibrils and a qualitative change in Zinn’s zonule (Figure 1C).

### 2.2. Observation of Inflammatory Changes in Zinn’s Zonule Using IF and RT-qPCR

**Figure 2:**
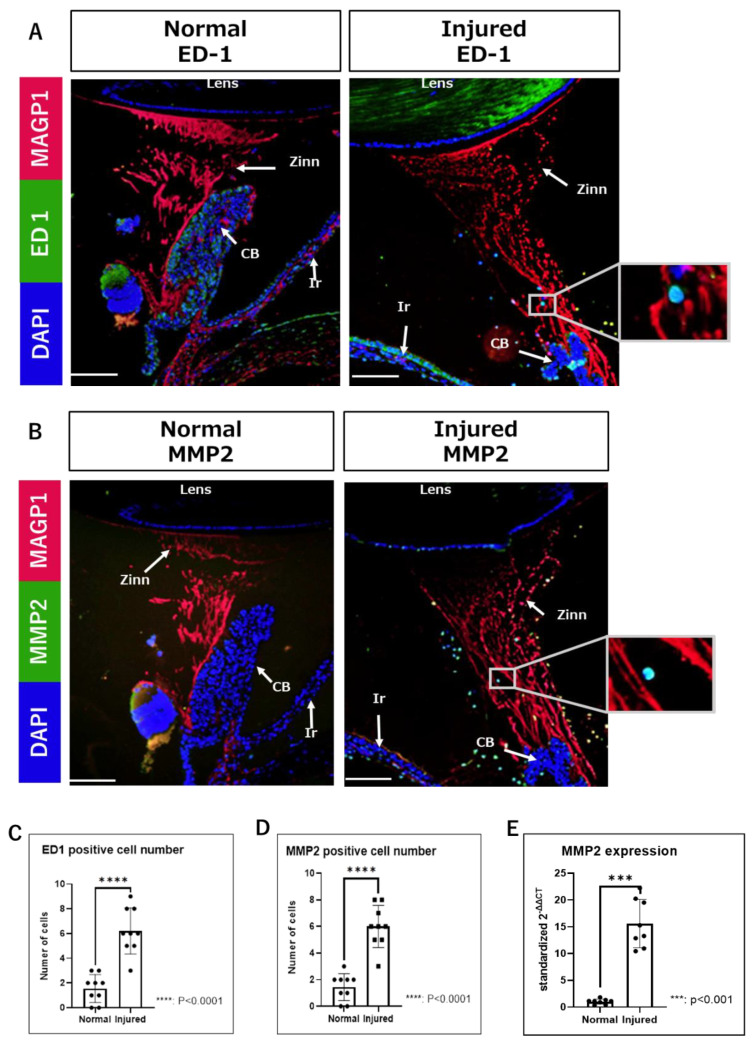
Increased infiltration of ED1-positive and MMP2-positive cells in Zinn’s zonule of the injured groups. (**A**,**B**) Immunofluorescence observations (magnified 100×) were performed on paraffin sections. (**A**) Nuclei were identified using DAPI (blue), macrophages using ED-1 (green), and Zinn’s zonule using anti-MAGP1 (red). (**B**) Nuclei were identified using DAPI (blue), MMP2-expressing cells using anti-MMP2 (green), and Zinn’s zonule using anti-MAGP1 (red). The cells in the white boxes in the figure are magnified. (**C**,**D**) Nucleated cells infiltrating Zinn’s zonule are increased in the injured group compared to the normal group. Both ED-1-positive and MMP2-positive cells are significantly increased in the injured group compared to the normal group. Four rat specimens were used for the normal and the injured groups, and three 5 μm sections were imaged from each specimen for both groups. The total number of observation areas was 9 due to the reason of the structural breakdown of Zinn’s zonule. We observed Zinn’s zonule in two locations in each section. (**E**) RT-qPCR was performed two times using RNA extracted from paraffin-fixed sections, showing significantly increased expression of MMP2 in the injured group compared to the normal group. A total of 100 thin sections of 10 µm thickness for each group were used for the experiments. Zinn: Zinn’s zonule, CB: ciliary body, Ir: iris. Scale bar: 100 μm.

Next, we performed immunofluorescence (IF) analysis using a paraffin-embedded tissue section. No effect of gelatin, such as the enhancement of background fluorescence, was observed during fluorescence microscopy. Nucleated cells migrating on Zinn’s zonule are found in both the normal and injured groups (Figure 2A). Infiltrating cells were stained for CD68 (ED-1) and matrix metalloproteinase 2 (MMP2). ED-1 was used as a marker for macrophages. Next, the relationship between ED-1-positive macrophages and MMP2 expression in them upon inflammatory changes in Zinn’s zonule was investigated.

Compared to the normal group, ED-1-positive and MMP2-positive infiltrating cells are significantly increased in the injured group (Figure 2B,C). In addition, the bundle structure of Zinn’s zonule is disrupted in the injured group (Figure 2A).

**Figure 3:**
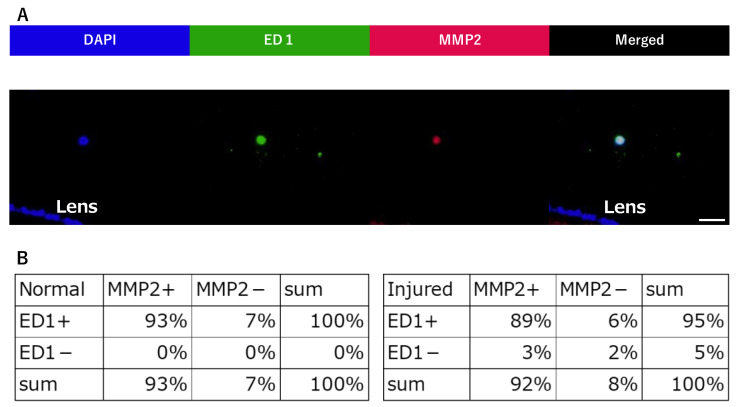
Quantification of ED1-positive and MMP2-positive cells infiltrated in Zinn’s zonule. (**A**) This image is in the injured group. Immunofluorescence observations (magnified 400×) were performed on paraffin sections. Nuclei were identified using DAPI (blue), macrophages using anti-ED-1 (green), and MMP2-positive cells using anti-MMP2 (red). Nucleated cells near the lens were counted and imaged to determine whether they were ED1-positive or MMP2-positive. The breakdown was counted. (**B**) The number of single-positive cells and double-positive cells for ED1 and MMP2 were counted and represented in the table. Scale bar: 20 μm. Four rat specimens were used for the normal and the injured groups, and three 5 μm sections were imaged from each specimen, for both groups. We observed Zinn’s zonule in two locations in each section.

Sufficient amounts of RNA could be extracted from each sample. The mRNA expression levels of MMP2 are significantly increased in the injured group compared to the normal group (Figure 2D). Furthermore, after counting the cell classifications, we observe that many ED-1-positive cells express MMP2 (Figure 3A,B). Altogether, these results suggests that macrophages expressing MMP2 are associated with the morphological changes in Zinn’s zonule.

### 2.3. Ultrastructural Observation of Inflammatory Changes in Zinn’s Zonule Using LV-SEM

Ultrastructural analysis was performed on a paraffin-embedded section using low-vacuum scanning electron microscopy (LV-SEM). The heavy metal enhancement method [7] was employed, which allows for the detection of areas positively stained by immunohistochemistry (IHC), as these are enhanced in LV-SEM.

**Figure 4:**
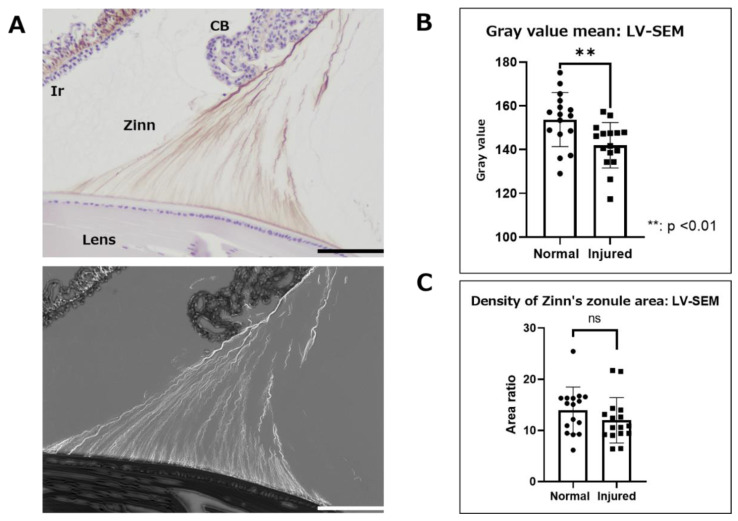
Immunohistochemical observation with LV-SEM analysis using paraffin-embedded specimens. Zinn’s zonules were labeled with anti-MAGP1 antibody and the heavy metal enhancement method was used to potentiate signals derived from DAB staining. Observations of Zinn’s zonule were made at low magnification (300×) to evaluate overall changes. (**A**) This image is in the normal group. The DAB-stained areas were clearly stained white by heavy metal staining in LV-SEM observations. (**B**) Zinn’s zonule area and gray value, a measure of color density, were measured using the color range selection function of Adobe Photoshop. (**C**) The area enclosed by the lens capsule, the anterior and posterior branches of Zinn’s zonule, and the area occupied by Zinn’s zonule were also used to measure density. Four specimens were used for the normal and injured groups and two 5 μm sections were made from each specimen. The total number of observation areas was 16. We observed Zinn’s zonule in two locations of each section. LV-SEM: low-vacuum scanning electron microscopy, Zinn: Zinn’s zonule, CB: ciliary body, Ir: iris. Scale bar: 100 μm.

First, Zinn’s zonule was observed at low magnification (300×) to assess overall changes (Figure 4). IHC with LV-SEM observations were compared with optical microscopy observations to confirm the consistency of the positive signal in IHC and LV-SEM (Figure 4A). The same areas that were stained with 3,3’-diaminobenzidine (DAB) using MAGP1, meaning they could be observed by LV-SEM as an enhanced signal with higher brightness. We then compared the normal and the injured groups for gray values, which is a measure of the color intensity of stained areas. A comparison of gray values was used to estimate differences in the amount of MAGP1. Furthermore, we estimated the relative area of Zinn’s zonule by comparing the ratio of the area enclosed by the anterior and posterior branches as well as the lens (Figure 4C). The results of the measurement of MAGP1 intensity at low magnification show a significant difference between the normal group and the injured group in color density (Figure 4B). On the other hand, the area ratio does not show any significant difference between the two groups (Figure 4C). These results suggest that inflammatory changes in Zinn’s zonule are not quantitative but qualitative, and result in a decrease in MAGP1.

**Figure 5:**
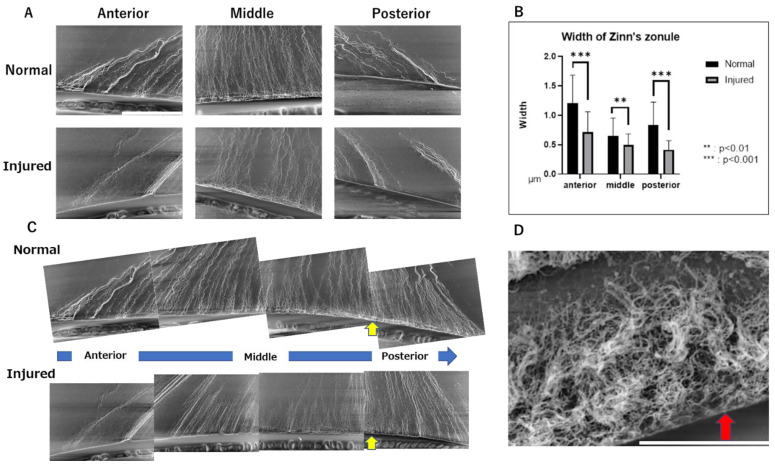
Ultrastructural observations of Zinn’s zonules labeled with anti-MAGP1 antibody using LV-SEM. (**A**) Observations of Zinn’s zonule were made at 1500× magnification with LV-SEM to evaluate changes near the lens. Zinn’s zonule was observed in the anterior and posterior branches and in their center. Scale bar: 50 μm. (**B**) The width of Zinn’s zonule was measured in 10 random locations on each slide. Compared to the normal group, Zinn’s zonule is significantly thinner in all locations in the injured group. The anterior and posterior cecal branches show even more significant differences. (**C**) Each of the 1500× magnified images was composited as a series and observed. On the lens capsule in the area noted by the yellow arrow, a structure not observed in the anterior capsule branch attachment area is observed. This is thought to be the fibrillar girdle. (**D**): Observations of Zinn’s zonule were made at 8000× magnification with LV-SEM. The red arrow indicates a mesh structure on the lens capsule that is thought to be a fibrillar girdle. Scale bar: 10 μm. Four specimens were used for the normal and injured groups and three 5 μm sections were made from each specimen. We observed Zinn’s zonule in two locations of each section. LV-SEM: low-vacuum scanning electron microscopy.

Next, we observed these samples at higher magnification (1500×) to assess changes in the ultrastructure of Zinn’s zonule in detail. We observed the anterior, middle, and posterior branches in the area near the lens attachment site (Figure 5A) and measured the width of each Zinn’s zonule (Figure 5B). To observe the overall trend, images were connected from the anterior to the posterior branch (Figure 5C). Under high magnification, Zinn’s zonule is significantly thinner throughout the eyes with alkaline injury and the bundled structure of Zinn’s zonule is also disrupted in the injured group (Figure 5A–C).

**Figure 6:**
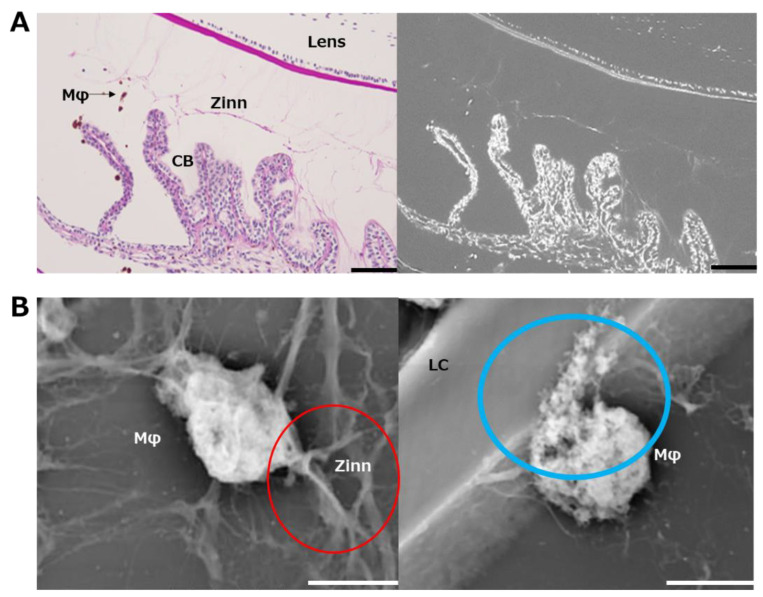
Ultrastructural observation of macrophages infiltrated in Zinn’s zonule using immunohistochemistry with LV-SEM. (**A**) Macrophages were labeled with ED-1. Periodic acid–Schiff (PAS) staining was used for post-staining. Areas stained with DAB are stained white by heavy metal staining in LV-SEM at 300×. Zinn’s zonule and other tissues are also stained white. Zinn: Zinn’s zonule, CB: ciliary body. Scale bar: 100 μm. (**B**) Macrophages were observed under LV-SEM at 10,000×. Zinn’s zonule and macrophages, which are difficult to distinguish at low magnification, become distinguishable at higher magnification due to the color density and contrast. In the red-circled area, a macrophage is observed pulling on Zinn’s zonule. In the blue-circled area, a macrophage is observed to be caving into the lens capsule. Zinn: Zinn’s zonule, LC: lens capsule. Scale bar: 5 μm.

In addition, a structure thought to be a fibrillar girdle [8,9] was identified at the lens capsule attachment area of the posterior capsule branch (Figure 5C). The mesh structure could be seen in the highest magnified image (Figure 5D). Finally, we performed LV-SEM observation for the ED1-stained IHC specimen to visualize the infiltrating macrophages on Zinn’s zonule (Figure 6A). We observe that the macrophages migrate along Zinn’s zonule and infiltrate into it to form various shapes (Figure 6B). Macrophage migration itself is also suggested to be a cause of damage to Zinn’s zonule.

## 3. Discussion

To observe macroscopic changes in Zinn’s zonule, gelatin-embedded specimens were prepared separately from paraffin sections to be used for IHC with LV-SEM, frozen sections were used for IF microscopy, and specimens were used for molecular biological examination. In addition, paraffin-fixed and frozen samples were analyzed as sections of 5 µm and 20 µm thickness, respectively. Due to the diverse locations of the tissue sections, it is difficult to draw precise conclusions. Furthermore, the results of RT-qPCR are ambiguous.

The purpose of this study was to establish an experimental and observational system to observe the effects of anterior ocular inflammation on Zinn’s zonule. Although there are reports of macroscopic observation with gelatin embedding [10], it was necessary to use a separate specimen for each of those microscopic morphological changes and cellular observations. In addition, IHC with LV-SEM has been used in the renal region [11,12,13], but there are few reports of its use for observation of the eye [14,15]. In this study, we succeeded in preparing paraffin-substitutable specimens from gelatin-embedded specimens by using low-melting-point gelatin. The paraffin-substitutable specimens enabled us to perform IF observation, IHC with LV-SEM observation, and RT-qPCR on the same specimens.

Our method enabled us to observe the changes caused by strong anterior ocular inflammation due to corneal alkali injury, with the same specimens, and seamlessly from the macro to the micro level. Gelatin-embedded specimens allowed quantitative observation of macroscopic changes in Zinn’s zonule. IF observations allowed us to analyze the characteristics of cells migrating on Zinn’s zonule. LV-SEM allowed ultrastructural observation of macroscopic morphological changes. In addition, the relationship between pathological and molecular biological changes in conventional paraffin sections and RT-qPCR can be evaluated more strictly by using this method.

Macroscopic and microscopic observation of the injured eyes show that Zinn’s zonule become thin and faded, suggesting a decrease in MAGP1, a binding protein of fibrillin 1 (Figure 1C and Figure 4B). In addition, the structure of Zinn’s zonule is disrupted in the injured eyes (Figure 5A,B). These results suggest that inflammation originating from the cornea reduces MAGP1 in Zinn’s zonule, which results in the thinning of Zinn’s zonule. IF observation also show an increase in the number of cells infiltrating Zinn’s zonule. Most infiltrating cells are MMP2-positive macrophages, and RT-qPCR examination also shows increased expression of MMP2. In addition, macrophages are observed migrating with traction on Zinn’s zonule.

MMP2 is expressed in many tissues in the eye [16]. There are also reports that MMP2 and macrophages are associated with changes in the sclera [17]. MMP2 has been reported to proteolyze microfibrils in in vitro experiments [18,19,20]. These results suggest that Zinn’s zonule is disassembled by MMP2-expressing macrophages that infiltrate the zonule as a result of inflammatory responses. In addition, from the observation that macrophages are migrating by pulling on Zinn’s zonule, damage due to the migration itself is also expected. A tissue called the fibrillar girdle was also identified, and LV-SEM images revealed its presence at the attachment of the posterior capsule branch, as previously reported [8,9].

This experimental setup is expected to be suitable for observing the corneal endothelium and retina, as it is suitable for obtaining an overall view of structures that are difficult to observe with flat images. Specifically, a genetic mechanism of corneal endothelial cell regeneration can be better related to pathological findings, and the macroscopic progression of retinal degenerative disease can be more strictly related to the mechanism of the disease. Zinn’s zonule is a non-vascular tissue composed of an extracellular matrix, and cells migrate on Zinn’s zonule from the ciliary body to the lens. Furthermore, the structure is isolated from surrounding tissues. These features make Zinn’s zonule a suitable tissue for observing cell migration. It is also expected to be applicable as a model for the mechanisms of macrophage infiltration in response to inflammatory changes in various tissues and their treatment methods.

## 4. Materials and Methods

### 4.1. Animals and Alkali Burn Model

Eight-week-old male *Wistar rats* (Sankyo Laboratory Service, Tokyo, Japan) were used for all experiments. All rats were maintained in a filtered-air laminar flow and were provided food ad libitum. All animal experiments were conducted in compliance with the Experimental Animal Ethics Review Committee of Nippon Medical School (approval number: 2019-005, 1 April 2020), and all procedures conformed with the requirements of the Association for Research in Vision and Ophthalmic and Visual Research. The endpoint was day 7 in both the normal and injured groups (*n* = 4 for each). The corneal alkali burn injury was made by placing round filter paper (3.2 mm diameter), soaked with 1M NaOH (0.3 μL), on the central cornea for 1 min, as the rats were maintained under isoflurane-based anesthesia. After that, the cornea was immediately rinsed with a 40-mL saline solution. Rats were sacrificed under general anesthesia. Perfusion fixation was performed with 4% paraformaldehyde (PFA)/ phosphate-buffered saline (PBS).

The eyeball was removed from the rats using a 29-G needle. It was inserted through the posterior part of the eyeball for intraocular pressure control, and 0.01 mL of 4% PFA/PBS was injected into the eyeball. The eye was then fixed with 4% PFA/PBS overnight; after that, the retinal side was removed near the pars plana line.

### 4.2. Gel- and Paraffin-Embedding of Eyes

**Figure 7:**
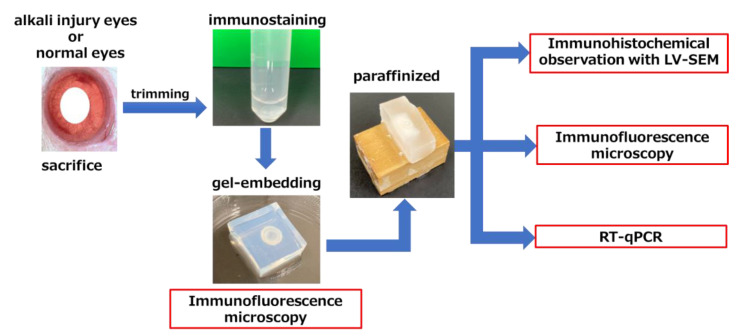
Schematic representation of a multiple-observation model of a single specimen. Each group contained four specimens. At each endpoint, the eyes were fixed after sacrificing the rats and trimmed to retrieve the anterior eye only. Zinn’s zonule was fluorescently stained with MAGP1, a component of Zinn’s zonule, and embedded in a gel. After initial observations, the gelatin in these sections was replaced with paraffin, and 5 µm sections were made for LV-SEM and fluorescent staining. LV-SEM, low-vacuum scanning electron microscopy; RT-qPCR, reverse transcription quantitative polymerase chain reaction.

Gel embedding was performed after IF staining. Gels were prepared using 0.5% agarose (Invitrogen, Waltham, MA) and 5% low-melting-point gelatin (Nitta Gelatin, Osaka, Japan) in PBS and melted by boiling it in a microwave/oven. The gel was then cooled to 40 °C and poured into Petri dishes [10,21]. The trimmed whole eye was embedded with the corneal side up and adjusted to be in a horizontal position (Figure 7). After macroscopic observation, gel-embedded specimens were incubated in 4% PFA/PBS overnight and the gelatin was replaced with paraffin. Paraffin-embedded specimens were sectioned into 5 µm slices for IF and LV-SEM, and sliced to 10 µm for RT-qPCR. Each section was de-paraffinized after being sliced. De-paraffinized slides used for IF and LV-SEM were incubated with a blocking solution (30% H_2_O_2_ + 100% methanol) to block endogenous peroxidase activity and washed with distilled water (DW) after incubation.

### 4.3. IF Analysis

IF staining was performed for gel-embedded specimens and paraffin sections. We used the following primary antibodies: (1) goat anti-mouse MAGP1 (R&D Systems, Minneapolis, MN; to detect Zinn’s zonule [22,23]); (2) mouse anti-rat ED-1 (BMA, Nagoya, Japan; to detect infiltrating macrophages); (3) rabbit anti-rat MMP2 (Abcam, Cambridge, UK). DAPI was used for nuclear counterstaining. The following secondary antibodies were used: (1) donkey anti-goat Alexa Fluor (AF)-488 (Abcam); (2) donkey anti-mouse AF488 (Abcam); (3) donkey anti-rabbit AF488 (Abcam); (4) donkey anti-rabbit AF594 (Abcam); (5) donkey anti-goat AF594 (Abcam). Fluorescent images were obtained using Olympus BX53 (Olympus, Tokyo, Japan) at 40×magnification for Figure 1, 100× for Figure 2, and 400× for Figure 3.

#### 4.3.1. IF Staining of Gel-Embedded Specimens

Fixed eyes were rinsed with PBS and then incubated in 0.1% Triton-X/5% bovine serum albumin (BSA) in PBS for 1 h at room temperature. Anti-MAGP1 antibody (1:1000 in PBS) was used and incubated overnight at room temperature. Secondary antibody and nuclear counterstain, AF488 (diluted 1:200 in PBS) and DAPI (diluted 1:1000 in PBS), respectively, were used and incubated for 2 h at room temperature.

#### 4.3.2. Paraffin Sections

We used the Opal 4-Color Manual IHC Kit (Akoya Biosciences, Marlborough, MA). Slides were incubated with 20 mL AR6 (included in Opal kit) diluted with 180 mL DW in a 200-W microwave for 20 min followed by washes with Tris-buffered saline with Tween 20 (TBST) for 3 min after cooling down to room temperature. Slides were incubated with Opal Antibody Diluent/Block (included in Opal kit) for 10 min at room temperature, which was then carefully removed. The following primary antibodies were used: MAGP1 (1:1000), ED1 (1:500), and MMP2 (1:250). Each antibody was diluted in the same TBST solution. Slides were incubated with primary antibody solution for 60 min and washed 3 times with TBST for 3 min each. The following secondary antibodies and nuclear counterstain were used: AF488 (1:500), AF594 (1:500), and DAPI (1:1000). Slides were incubated with secondary antibody solution for 30 min and washed once with TBST for 3 min. Then, Vector True View was used to suppress autofluorescence, and after washing with TBS, the slides were sealed with VECTASHIELD mounting medium H-1400.

### 4.4. IHC with LV-SEM Analysis

Sections were examined under light microscopy and LV-SEM (TM3030 Plus tabletop microscope; Hitachi High Technologies Corp., Tokyo, Japan) using an acceleration voltage of 15 kV with 30 Pa for the backscattered electron detector. We used MAGP1 and ED-1 as primary antibodies and visualized antigens with DAB. MAGP1-stained slides were stained with Meyer hematoxylin staining, and ED-1-stained slides were stained with periodic acid–Schiff staining. For LV-SEM observation, we used Ag+Au+Pt staining. The staining methods [10] used are as follows: Ag-enhanced specimens were incubated for 20 min at 60 °C in Ag solution containing 25 mL of 3% methenamine, 2.5 mL of 5% silver nitrate, 22.5 mL of DW, and 2.5 mL of 5% sodium tetraborate. Later, specimens were rinsed with deionized water and placed in deionized water. Au-enhanced specimens were incubated in 0.25% Au for 5 min at room temperature. Then, specimens were rinsed with deionized water and placed in deionized water. Pt-enhanced specimens were incubated in Pt with 25% ammonia solution for 10 min at room temperature.

### 4.5. RT-qPCR Analysis

Each specimen was sliced into 100 slices of 10 µm thickness each and pooled together for analysis. After deparaffinization, specimens were carefully peeled from the slides using a razor and collected. RNA was extracted from specimens using the RNeasy FFPE Kit (QIAGEN, Hilden, Germany) in accordance with the manufacturer’s instructions [24]. The concentration and purity of the extracted RNA was measured using a NanoDrop ND-1000 V3.2.1 Spectrophotometer (NanoDrop Technologies, Wilmington, DE, USA). cDNAs were synthesized from total RNA using the High-Capacity cDNA Reverse Transcription kit (Thermo Fisher Scientific, Waltham, MA, USA) in accordance with the manufacturer’s instructions.

Target genes were amplified (2 min at 50 °C, 10 min at 95 °C, and 45 cycles of denaturation at 95 °C for 15 s and annealing at 60 °C for 60 s) using the Quant Studio 3 Real-Time PCR System (Thermo Fisher Scientific), THUNDERBIRD SYBR qPCR Mix (TOYOBO, Osaka, Japan), and specific primers. The primers used in this experiment are as follows: β actin (forward: 5′-GCAGGAGTACGATGAGTCCG-3′, reverse: 5′-ACGCAGCTCAGTAACAGTCC-3′), MMP2 (forward: 5′-ACACTTTCTATGGCTGCCCC-3′, reverse: 5′-CCGGTCATAATCCTCGGTGG-3′). The results were calculated by the ΔΔCT method [25].

### 4.6. Image Analysis

Image analysis was performed using Photoshop (Version 24.0; Adobe, San Jose, CA, USA) for signal intensity and width measurement. The black balance was set at the same value for both the normal and the injured group. The color range selection was made without including the background signal based on the fluorescent area of Zinn’s zonule for analysis in Photoshop. The background signal was eliminated as much as possible by these two processes.

### 4.7. Statistical Analysis

All statistical analyses were performed using non-parametric t-tests. A p-value less than 0.05 was considered statistically significant. All analyses were performed using GraphPad Prism software (Version 8.4.2; GraphPad Software, San Diego, CA, USA). All results are expressed as the mean ± standard error.

## 5. Conclusions

In this study, we designed a new experimental system that allows macroscopic observation using gelatin-embedded specimens, microscopic observation using paraffin-embedded specimens, immunohistology, and gene expression analysis to be performed using a single source specimen.

This experimental system is useful for observing the relationship between macrophages and Zinn’s zonule. Using this system, we could observe morphological changes in the specimen and acquire gene expression and immunohistological data of the same specimen. We believe that, in the future, this technique can be applied to various specimens to study their macro- and micro-aspects at the same time.

## Data Availability

The data presented in this study are available on reasonable request from the corresponding author.

## References

[1] Ascaso F.J., Huerva V., Grzybowski A. (2015). Epidemiology, etiology, and prevention of late IOL-capsular bag complex dislocation: Review of the literature. J. Ophthalmol..

[2] Ladewig M.S., Robinson P.N., Neumann L.M., Holz F.G., Foerster M.H. (2006). Ocular manifestations and surgical results in patients with Marfan syndrome. Ophthalmologe.

[3] Canavan Y.M., Archer D.B. (1982). Anterior segment consequences of blunt ocular injury. Br. J. Ophthalmol..

[4] Shingleton B.J., Neo Y.N., Cvintal V., Shaikh A.M., Liberman P., O’Donoghue M.W. (2017). Outcome of phacoemulsification and intraocular lens implantation in eyes with pseudoexfoliation and weak zonules. Acta Ophthalmol..

[5] Tao L.W., Hall A. (2015). In-bag dislocation of intraocular lens in patients with uveitis: A case series. J. Ophthalmic Inflamm. Infect..

[6] DeDreu J., Bowen C.J., Logan C.M., Pal-Ghosh S., Parlanti P., Stepp M.A., Menko A.S. (2020). An immune response to the avascular lens following wounding of the cornea involves ciliary zonule fibrils. FASEB J..

[7] Arai Y., Takeuchi K., Hatanaka S., Ishikawa A., Inoue T., Takakuma S., Kajimoto Y., Toda E., Kunugi S., Terasaki M. (2022). Heavy metal enhancement technique for diaminobenzidine in immunohistochemistry enables ultrastructural observation by low-vacuum scanning electron microscopy. J. Histochem. Cytochem..

[8] Shi Y., Tu Y., De Maria A., Mecham R.P., Bassnett S. (2013). Development, composition, and structural arrangements of the ciliary zonule of the mouse. Investig. Ophthalmol. Vis. Sci..

[9] Shi Y., Jones W., Beatty W., Tan Q., Mecham R.P., Kumra H., Reinhardt D.P., Gibson M.A., Reilly M.A., Rodriguez J. (2021). Latent-transforming growth factor beta-binding protein-2 (LTBP-2) is required for longevity but not for development of zonular fibers. Matrix Biol..

[10] Bassnett S. (2019). A method for preserving and visualizing the three-dimensional structure of the mouse zonule. Exp. Eye Res..

[11] Miyazaki H., Uozaki H., Tojo A., Hirashima S., Inaga S., Sakuma K., Morishita Y., Fukayama M. (2012). Application of low-vacuum scanning electron microscopy for renal biopsy specimens. Pathol. Res. Pract..

[12] Song J.Y., Saga N., Kawanishi K., Hashikami K., Takeyama M., Nagata M. (2020). Bidirectional, non-necrotizing glomerular crescents are the critical pathology in X-linked Alport syndrome mouse model harboring nonsense mutation of human COL4A5. Sci. Rep..

[13] Fujimaru T., Kawanishi K., Mori T., Mishima E., Sekine A., Chiga M., Mizui M., Sato N., Yanagita M., Ooki Y. (2021). Genetic background and clinicopathologic features of adult-onset nephronophthisis. Kidney Int. Rep..

[14] Arima T., Uchiyama M., Shimizu A., Takahashi H. (2020). Observation of corneal wound healing and angiogenesis using low-vacuum scanning electron microscopy. Transl. Vis. Sci. Technol..

[15] Ikebukuro T., Arima T., Kasamatsu M., Nakano Y., Tobita Y., Uchiyama M., Terashima Y., Toda E., Shimizu A., Takahashi H. (2023). Disulfiram ophthalmic solution inhibited macrophage infiltration by suppressing macrophage pseudopodia formation in a rat corneal alkali burn model. Int. J. Mol. Sci..

[16] Sivak J.M., Fini M.E. (2002). MMPs in the eye: Emerging roles for matrix metalloproteinases in ocular physiology. Prog. Retin. Eye Res..

[17] Zhao F., Wu H., Reinach P.S., Wu Y., Zhai Y., Lei Y., Ma L., Su Y., Chen Y., Li F. (2020). Up-regulation of matrix metalloproteinase-2 by scleral monocyte-derived macrophages contributes to myopia development. Am. J. Pathol..

[18] Shiroto Y., Saga R., Yoshino H., Hosokawa Y., Isokawa K., Tsuruga E. (2021). Matrix metalloproteinase-2 activated by ultraviolet-B degrades human ciliary zonules. Acta Histochem. Cytochem..

[19] Ashworth J.L., Murphy G., Rock M.J., Sherratt M.J., Shapiro S.D., Shuttleworth C.A., Kielty C.M. (1999). Fibrillin degradation by matrix metalloproteinases: Implications for connective tissue remodelling. Biochem. J..

[20] Kawagoe M., Tsuruga E., Oka K., Sawa Y., Ishikawa H. (2013). Matrix metalloproteinase-2 degrades fibrillin-1 and fibrillin-2 of oxytalan fibers in the human eye and periodontal ligaments in vitro. Acta. Histochem. Cytochem..

[21] Ushida K., Asai N., Uchiyama K., Enomoto A., Takahashi M. (2018). Development of a method to preliminarily embed tissue samples using low melting temperature fish gelatin before sectioning: A technical note. Pathol. Int..

[22] Bassnett S. (2021). Zinn’s zonule. Prog. Retin. Eye Res..

[23] Mecham R.P., Gibson M.A. (2015). The microfibril-associated glycoproteins (MAGPs) and the microfibrillar niche. Matrix Biol..

[24] LeVaillant C.J., Sharma A., Muhling J., Wheeler L.P., Cozens G.S., Hellström M., Rodger J., Harvey A.R. (2016). Significant changes in endogenous retinal gene expression assessed 1 year after a single intraocular injection of AAV-CNTF or AAV-BDNF. Mol. Ther. Methods Clin. Dev..

[25] Livak K.J., Schmittgen T.D. (2001). Analysis of relative gene expression data using real-time quantitative PCR and the 2(-Delta Delta C(T)) method. Methods.

